# Double-slit holography—a single-shot lensless imaging technique

**DOI:** 10.1038/s41598-024-62785-7

**Published:** 2024-05-31

**Authors:** Flavio Wicki, Tatiana Latychevskaia

**Affiliations:** 1 Physics Department, University of Zurich, Winterthurerstrasse 190, 8057 Zurich, Switzerland; 2 Paul Scherrer Institute, Forschungsstrasse 111, 5232 Villigen, Switzerland

**Keywords:** Imaging and sensing, Applied optics, Microscopy

## Abstract

In this study, we propose a new method for single-shot, high-resolution lensless imaging called double-slit holography. This technique combines the properties of in-line and off-axis holography in one single-shot measurement using the simplest double-slit device: a plate with two apertures. In double-slit holography, a plane wave illuminates the two apertures giving rise to two spherical waves. While diffraction of one spherical wave from a sample positioned behind the first aperture (the object aperture) provides the object wave, the other spherical wave diffracted from the second (reference) aperture provides the reference wave. The resulting interference pattern in the far-field (hologram) combines the properties of an in-line (or Gabor-type) hologram and an off-axis hologram due to the added reference wave from the second aperture. Both the object and reference waves have the same intensity, which ensures high contrast of the hologram. Due to the off-axis scheme, the amplitude and phase distributions of the sample can be directly reconstructed from the hologram, and the twin image can be easily separated. Due to the object wave being the same as in-line holography with a spherical wave, imaging at different magnifications is similarly done by simply adjusting the aperture-to-sample distance. The resolution of the reconstructed object is given by the numerical aperture of the optical setup and the diameter of the reference aperture. It is shown both by theory and simulations that the resolution of the reconstructed object depends on the diameter of the reference wave aperture but does not depend on the diameter of the object aperture. Light optical proof-of-concept experiments are provided. The proposed method can be particularly practical for X-rays, where optical elements such as beam splitters are not available and conventional off-axis holography schemes cannot be realised.

## Introduction

The original holography scheme proposed by Dennis Gabor—in-line holography provides the highest possible resolution of the reconstructed object due to the maximal possible numerical aperture of the optical scheme, but the reconstructed object distribution is contaminated by the so-called twin image^1,2^, which can be eliminated in post-analysis by applying iterative reconstruction methods^3^. An experimental solution to the twin image problem is off-axis holography, where the object and reference beams are tilted relatively to each other, which allows for spatial separation of the object and its twin reconstruction^4,5^. An experimental realization of off-axis holography, however, requires additional optical elements (beam splitters and mirrors), which also increases the requirements for the coherence of the probing wave. Off-axis holography is realized with light by typically using an interferometric scheme^6^, or with electrons by using a biprism^7^. In addition, off-axis holography provides lower resolution than in-line holography because, during the reconstruction procedure, a sideband is cut out in the Fourier spectrum of the hologram; this cutout acts as a low-pass filter, thus removing high-resolution information. Off-axis holography allows phase recovery by a simple reconstruction procedure from a single-shot intensity measurement. There are many other types of holography that allow phase recovery, however, they require more than one intensity measurement. A few examples include: phase-shifting holography^8,9^, non-iterative Kramers Kronig method^10^, three-dimensional wavefront reconstruction from two intensity measurements by iterative phase retrieval^11^, and phase retrieval based on combining in-line and off-axis holograms^12^.

Methods that combine the advantages of in-line and off-axis holographic schemes in one single-shot hologram were previously suggested: Fourier transform holography (FTH)^13,14^ and Fresnel coherent diffraction imaging^15,16^. FTH is the most popular method in holography with X-rays. Similar to off-axis holography, it uses a reference wave that originates from an aperture located next to the object. FTH employs an easy reconstruction procedure by simply calculating the Fourier transform (FT) of the hologram. However, the main drawback of FTH is the issue related to the resolution and intensity of the reference wave. The resolution in FTH is given by the diameter of the reference aperture. Thus, the diameter of the reference aperture should be minimised to achieve better resolution. At the same time, a smaller diameter of the reference aperture results in a lower intensity of the reference beam, but the intensities of the object and reference waves should be of the same magnitude to ensure a high contrast of the interference pattern in the hologram.

The idea of combining Gabor-type holography and off-axis holography in single-shot intensity measurement by using two mutually coherent sources has been previously explored; however, the proposed experimental schemes require sophisticated experimental arrangements. Fuhse et al. presented an off-axis holography experiment based on coherent cone beams emitted from a pair of X-ray waveguides^17^. Serabyn et al. described a compact setup for lensless off-axis holographic microscopy using a pair of GRIN lenses^18,19^. Most recently, Arcab et al. demonstrated holography using two wavefronts emerging from two fibers^20^.

In this study, we propose double-slit holography, which is a single-shot holography method that allows recording of one hologram that combines the properties of an in-line and off-axis hologram in one single-shot measurement by using the simplest device: a metal plate with two apertures, which we name a double-slit device. This type of holography combines the high numerical aperture and flexible magnification of in-line holography with direct phase reconstruction of off-axis holography due to a tilted reference wave.

## Principle

### Hologram formation

**Figure 1:**
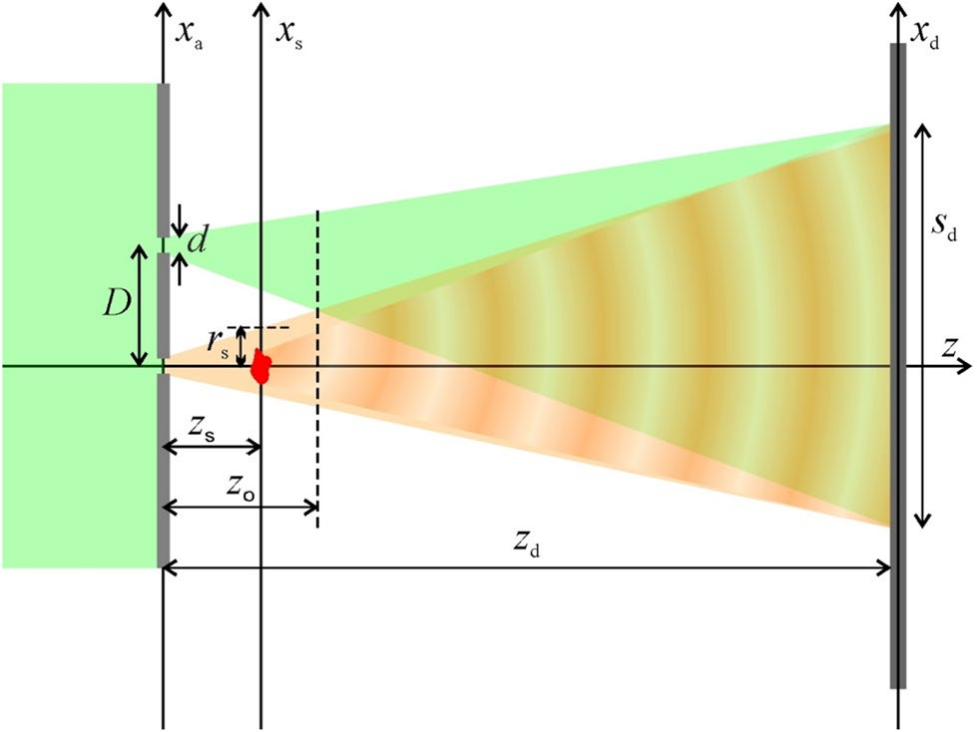
Experimental arrangement for double-slit holography (not drawn to scale). The optical axis is selected to be at the position of the aperture for the object wave for simplicity.

In double-slit holography, two apertures, each of diameter *d*, are separated by a distance of *D* and illuminated by a plane wave, as shown in Fig. 1. In this section, we derive the equations for the hologram's intensity, assuming that the apertures are infinitely small and described by delta-functions. The effect of the apertures' diameters is studied in detail in the sections below.

In the absence of an object, the two waves diffracted by the apertures produce an interference pattern with equidistant fringes in the detector plane (for simplicity, we consider a one-dimensional case here):

1 $$ I\left( {x_{{\text{d}}} } \right) = 2a_{0}^{2} I_{{0}} \left( {x_{{\text{d}}} } \right)\left[ {1 + \cos \left( {\frac{2\pi }{{\lambda z_{{\text{d}}} }}x_{{\text{d}}} D} \right)} \right], $$

where $I_{0} \left( {x_{{\text{d}}} } \right)$ is the diffraction pattern of a single aperture when illuminated by a plane wave of amplitude one, *x*_d_ is the coordinate in the detector plane, *a*_0_ is the amplitude of the illuminating wave, *z*_d_ is the distance between the aperture and detector planes, and *λ* is the wavelength. $I_{0} \left( {x_{{\text{d}}} } \right)$ is given by:

2 $$I_{0} \left( {x_{{\text{d}}} } \right) = \left| {U_{0} \left( {x_{{\text{d}}} } \right)} \right|^{2} ,$$

where $U_{0} \left( {x_{{\text{d}}} } \right)$ is the amplitude of the wave in the detector plane:

3 $$\tilde{U}_{0} \left( {x_{{\text{d}}} } \right) = 2u_{0} \left( {\frac{{2\pi ar_{{\text{d}}} }}{{\lambda z_{{\text{d}}} }}} \right)^{ - 1} J_{1} \left( {\frac{{2\pi ar_{{\text{d}}} }}{{\lambda z_{{\text{d}}} }}} \right)\exp \left( {\frac{i\pi }{{\lambda z_{{\text{d}}} }}r_{{\text{d}}}^{2} } \right) = U_{0} \left( {x_{{\text{d}}} } \right)\exp \left( {\frac{i\pi }{{\lambda z_{{\text{d}}} }}r_{{\text{d}}}^{2} } \right),$$

$u_{0} = - \frac{{\pi ia^{2} }}{{\lambda z_{{\text{d}}} }}$, $r_{{\text{d}}} = \sqrt {x_{{\text{d}}}^{2} + y_{{\text{d}}}^{2} }$ is the radial coordinate in the sample plane, $J_{1} \left( {...} \right)$ is the Bessel function of the first kind, and $a$ is the radius of the aperture ($d=2a$).

When an object is placed behind the object aperture at a distance $z_{{\text{s}}}$ (Fig. 1), the diffracted wave produces an in-line wavefront in the detector plane. The wavefront distribution of the diffracted wave in the detector plane is given by:

4 $$\begin{gathered} O\left( {x_{{\text{d}}} } \right) = - \frac{i}{{\lambda \left( {z_{{\text{d}}} - z_{{\text{s}}} } \right)}}\int {} u_{{{\text{prob}}}} \left( {x_{{\text{s}}} } \right)t\left( {x_{{\text{s}}} } \right) \exp \left[ {\frac{i\pi }{{\lambda \left( {z_{{\text{d}}} - z_{{\text{s}}} } \right)}} \left( {x_{{\text{s}}} - x_{{\text{d}}} } \right)^{2} } \right]{\text{d}}x_{{\text{s}}} \approx \hfill \\ \approx - \frac{i}{{\lambda z_{{\text{d}}} }}\exp \left( {\frac{i\pi }{{\lambda z_{{\text{d}}} }}x_{{\text{d}}}^{2} } \right)\int {} u_{{{\text{prob}}}} \left( {x_{{\text{s}}} } \right)t\left( {x_{{\text{s}}} } \right)\exp \left( { - \frac{2\pi i}{{\lambda z_{{\text{d}}} }}x_{{\text{s}}} x_{{\text{d}}} } \right){\text{d}}x_{{\text{s}}} = \hfill \\ = \exp \left( {\frac{i\pi }{{\lambda z_{{\text{d}}} }}x_{{\text{d}}}^{2} } \right)\tilde{O}\left( {x_{{\text{d}}} } \right), \hfill \\ \end{gathered}$$

where $u_{{{\text{prob}}}} \left( {x_{{\text{s}}} } \right)$ is the probing wave in the sample plane given by an expression similar to Eq. (3) (with $x_{{\text{d}}} \to x_{{\text{s}}} ,z_{{\text{d}}} \to z_{{\text{s}}}$), and $t\left( {x_{{\text{s}}} } \right)$ is the transmission function of the sample. Without the wavefront from the second (reference) aperture, the wavefront described by Eq. (4) creates an in-line hologram in the detector plane.

The wavefront diffracted from the reference aperture located at $\left( {x_{{{\text{a}},\text{ref}}} ,y_{{{\text{a}},\text{ref}}} } \right)$ is given by:

5 $$ \begin{aligned} R \left( {x_{{\text{d}}} } \right) = & - \frac{i}{{\lambda z_{{\text{d}}} }}\int\limits_{{{\text{aperture}}}} {} \exp \left\{ {\frac{i\pi }{{\lambda z_{{\text{d}}} }}\left[ {x_{{\text{d}}} - \left( {x_{{\text{a}}} - x_{{\text{a,ref}}} } \right)} \right]^{2} } \right\}{\text{d}}x_{{\text{a}}} = \hfill \\ = &U_{0} \left( {x_{{\text{d}}} } \right) {\exp \left( {\frac{i\pi }{{\lambda z_{{\text{d}}} }}x_{{\text{d}}}^{2} } \right)\exp \left( {\frac{2\pi i}{{\lambda z_{{\text{d}}} }}x_{{\text{d}}} x_{{\text{a,ref}}} } \right)},  \end{aligned} $$

where $U_{0} \left( {x_{{\text{d}}} } \right)$ is given by Eq. (3). The superposition of the two waves in the detector plane creates an off-axis hologram whose intensity distribution is described by:

6 $$I \left( {x_{{\text{d}}} } \right) = \left| {\tilde{O}\left( {x_{{\text{d}}} } \right) + U_{0} \left( {x_{{\text{d}}} } \right)\exp \left( {\frac{2\pi i}{{\lambda z_{{\text{d}}} }}x_{{\text{d}}} x_{{{\text{a}},\text{ref}}} } \right)} \right|^{2} ,$$

where $\tilde{O}\left( {x_{{\text{d}}} } \right)$ is given by Eq. (4). The interference pattern given by Eq. (6) describes a single-shot hologram that combines the properties of in-line and off-axis holograms.

### Parameters

For an aperture of diameter $d$, the radius of the diffracted beam (that is, the probing beam) in the sample plane is given by the first minimum of the Airy disks:

7 $$r_{{\text{s}}} = 1.22\frac{{\lambda z_{{\text{s}}} }}{d}.$$

The two intensity disks created by the two beams from the two apertures do not overlap when $r_{{\text{s}}} < D/2$, and they begin to overlap at a distance $z_{{\text{o}}}$ (illustrated in Fig. 1):

8 $$z_{{\text{o}}} = \frac{Dd}{{2.44\lambda }}$$

For example, for *λ* = 532 nm, the distance between two apertures *D* = 200 μm and *d* = 8 μm, Eq. (8) gives: *z*_o_ = 1.23 mm. The radius of the beam in the detector plane is also determined by the first minimum of the Airy disks:

9 $$r_{{\text{d}}} = 1.22\frac{{\lambda z_{{\text{d}}} }}{d}.$$

For λ = 532 nm, *D* = 200 μm, and *d* = 8 μm, Eq. (9) gives: *r*_d_ = 8.11 mm. The period of the interference pattern formed in the detector plane is given by:

10 $$T = \frac{{\lambda z_{{\text{d}}} }}{D},$$

and it should exceed 2 pixels of the detecting system to ensure the correct sampling of the interference pattern in the detector plane.

The magnification of the object image is the same as in in-line holography with spherical waves^2^ and calculated as^21^:

11 $$M = \frac{{z_{{\text{d}}} }}{{z_{{\text{s}}} }}.$$

A prominent example of using the magnification property of in-line (Gabor) holography with spherical waves is low-energy electron holography, where imaging of nano-sized objects such as single macromolecules or proteins at a magnification of about 10^5^ is achieved without the use of any lenses^22–24^.

### Reconstruction

The reconstruction of the sample distribution from its double-slit hologram begins with the same steps as for the reconstruction of an off-axis hologram:

- (1) FT of the hologram is calculated, and the result is the complex-valued spectrum of the hologram.
- (2) In the spectrum, a sideband at the carrying frequency is selected. The spectrum is shifted so that the selected sideband is in the center of the spectrum. The resulting spectrum is multiplied by a round mask, which sets the signal around the sideband to zero (low-pass filter).
- (3) Inverse FT of the distribution obtained in step (2) is calculated. The result is the complex-valued wavefront in the detector plane.
- (4) The complex-valued wavefront is propagated backward from the detector plane to the sample plane. The wavefront propagation can be calculated by using the algorithms applied in in-line holography with spherical waves^21^.

From the wavefront distribution in the sample plane, the quantitative absorption and phase shifting properties of the sample can be reconstructed, provided the distribution of the incident wave in the sample plane is known^25^. This distribution can be obtained by reconstructing a hologram recorded without the sample and using the same reconstruction steps (1)–(4).

### Resolution

Several parameters affect the final resolution of the reconstructed sample: the extent of the recorded hologram (*NA* of the optical setup), sampling, the finite extent of the spectrum in the FT domain, and the radius (or diameter) of the reference aperture. We do not consider resolution degradation due to coherence in this study, assuming full coherence of the waves. Below is a list of the resolution limits associated with each of the parameters.

(1) The resolution defined by the extent of the hologram (*NA* of the optical setup). This resolution is given by

12 $$R_{{{\text{NA}}}} = \frac{\lambda }{2NA} \approx \frac{{\lambda z_{{\text{d}}} }}{{s_{{\text{d}}} }},$$

where *s*_d_ is the extent (diameter) of the “effective hologram”—the extent of the interference pattern where the signal is higher than zero or noise level.

(2) The resolution due to digital sampling of the hologram. Numerical sampling of the hologram leads to a finite pixel size in the sample plane

13 $$R_{{\text{D}}} = \Delta_{{\text{s}}} = \frac{{z_{{\text{s}}} s_{{\text{d}}} }}{{Nz_{{\text{d}}} }}.$$

(3) The resolution due to the finite extent of the spectrum in FT domain. This resolution is defined by the size of the cutout region in the Fourier spectrum during the reconstruction procedure:

14 $$R_{{\text{F}}} = \frac{{z_{{\text{s}}} s_{{\text{d}}} }}{{2N_{{\text{a}}} z_{{\text{d}}} }},$$

where *N*_a_ is the radius of the cutout window (in pixels) in the Fourier domain.

(4) The resolution given by the radius of the reference aperture *a*. This resolution can be derived as follows. The wavefront diffracted by a finite-diameter aperture in the sample plane is given by:

15 $$u_{{{\text{prob}}}} \left( {x_{{\text{s}}} } \right) = - \frac{i}{{\lambda z_{s} }}\int\limits_{{{\text{aperture}}}} {} \exp \left[ {\frac{i\pi }{{\lambda z_{s} }}\left( {x_{s} - x_{{\text{a}}} } \right)^{2} } \right]{\text{d}}x_{{\text{a}}} ,$$

and the wavefront behind the sample is given by:

16 $$u\left( {x_{{\text{s}}} } \right) = u_{{{\text{prob}}}} \left( {x_{{\text{s}}} } \right)t\left( {x_{{\text{s}}} } \right).$$

The wavefront in the detector plane is given by:

17 $$\begin{gathered} U\left( {x_{{\text{d}}} } \right) = - \frac{i}{{\lambda \left( {z_{{\text{d}}} - z_{{\text{s}}} } \right)}} \int\limits_{{\phantom{a}}} {u_{{{\text{prob}}}} \left( {x_{{\text{s}}} } \right) t\left( {x_{{\text{s}}} } \right)} \exp \left[ {\frac{i\pi }{{\lambda \left( {z_{{\text{d}}} - z_{{\text{s}}} } \right)}}\left( {x_{{\text{s}}} - x_{{\text{d}}} } \right)^{2} } \right]{\text{d}}x_{{\text{s}}} \propto \hfill \\ \propto \left[ {u_{{{\text{prob}}}} \left( {x_{{\text{s}}} } \right)t\left( {x_{{\text{s}}} } \right)} \right] \otimes \exp \left( {\frac{i\pi }{{\lambda z_{{\text{s}}} }}x_{{\text{s}}}^{2} } \right). \hfill \\ \end{gathered}$$

When no object is present, then $t\left( {x_{{\text{s}}} } \right) = 1$ and this integral turns into the integral describing diffraction on a round aperture:

18 $$\begin{gathered} R\left( {x_{{\text{d}}} } \right) = - \frac{i}{{\lambda \left( {z_{{\text{d}}} - z_{{\text{s}}} } \right)}}\int\limits_{{\phantom{a}}} {\left\{ { - \frac{i}{{\lambda z_{s} }}\int\limits_{{{\text{aperture}}}} {} \exp \left[ {\frac{i\pi }{{\lambda z_{{\text{s}}} }}\left( {x_{{\text{s}}} - x_{{\text{a}}} } \right)^{2} } \right]{\text{d}}x_{{\text{a}}} } \right\}} \hfill \\ \exp \left[ {\frac{i\pi }{{\lambda \left( {z_{{\text{d}}} - z_{{\text{s}}} } \right)}}\left( {x_{{\text{s}}} - x_{{\text{d}}} } \right)^{2} } \right]{\text{d}}x_{{\text{s}}} = \hfill \\ = - \frac{i}{{\lambda z_{{\text{d}}} }}\int\limits_{{{\text{aperture}}}} {} \exp \left[ {\frac{i\pi }{{\lambda z_{{\text{d}}} }}\left( {x_{{\text{d}}} - x_{{\text{a}}} } \right)^{2} } \right]{\text{d}}x_{{\text{a}}} , \hfill \\ \end{gathered}$$

which is the reference wavefront from the reference aperture. Its distribution in the detector plane exhibits Airy disks. During the hologram formation, the reference wave is multiplied by the object wave $H \propto R^{*} O + RO^{*}$, and thus, the extent of the main maxima of the reference wave defines the hologram's effective size. The radial coordinate of the first zero of the Airy disks distribution is given by $r_{{\text{d}}} = {0.61\lambda z_{{\text{d}}} }/a$. It defines the extent of the hologram (*NA* of the optical setup), which in turn gives the following resolution of the reconstructed object:

19 $$R_{{\text{A}}} = 0.61\frac{{\lambda z_{{\text{d}}} }}{{r_{{\text{d}}} }} = a.$$

Similarly, the resolution in FTH is also given by the diameter of the reference aperture^14^. The simulation study of the effect of the object and reference aperture diameters on the resulting resolution is provided in the next section.

The resolution does not depend on the radius of the object aperture. This can be shown as follows. From Eq. (17), the object wave distribution is given by

20 $$O\left( {x_{{\text{d}}} } \right) \propto \int\limits_{{\phantom{a}}} {u_{{{\text{prob}}}} \left( {x_{{\text{s}}} } \right)t\left( {x_{{\text{s}}} } \right)} \exp \left[ { - \frac{2\pi i}{{\lambda \left( {z_{{\text{d}}} - z_{{\text{s}}} } \right)}}x_{{\text{s}}} x_{{\text{d}}} } \right]{\text{d}}x_{{\text{s}}} ,$$

which is the FT of the product of the probing wave and the sample transmission function. From Eq. (20), it can be seen that when the wavefront $u_{{{\text{prob}}}} \left( {x_{{\text{s}}} } \right)$ illuminates the entire object without reaching zero intensity, then the wavefront in the detector plane contains the entire information about the object without a loss of resolution. Another explanation can be provided by using the convolution theorem. Equation (20) can be rewritten as

21 $$O\left( {x_{{\text{d}}} } \right) \propto {\text{FT}}\left[ {u_{{{\text{prob}}}} \left( {x_{{\text{s}}} } \right)} \right] \otimes {\text{FT}}\left[ {t\left( {x_{{\text{s}}} } \right)} \right],$$

where ${\text{FT}}\left[ {u_{{{\text{prob}}}} \left( {x_{{\text{s}}} } \right)} \right]$ gives the aperture distribution and ${\text{FT}}\left[ {t\left( {x_{{\text{s}}} } \right)} \right]$ is the spectrum of the sample distribution. From Eq. (21), it follows that the object spectrum is not multiplied by the aperture function (which would be the same as applying a low-pass filter and lead to a loss of resolution), but instead it is convoluted with the aperture function. Thus, the resolution of the reconstructed object does not directly depend on the radius of the object aperture.

## Simulations

**Figure 2:**
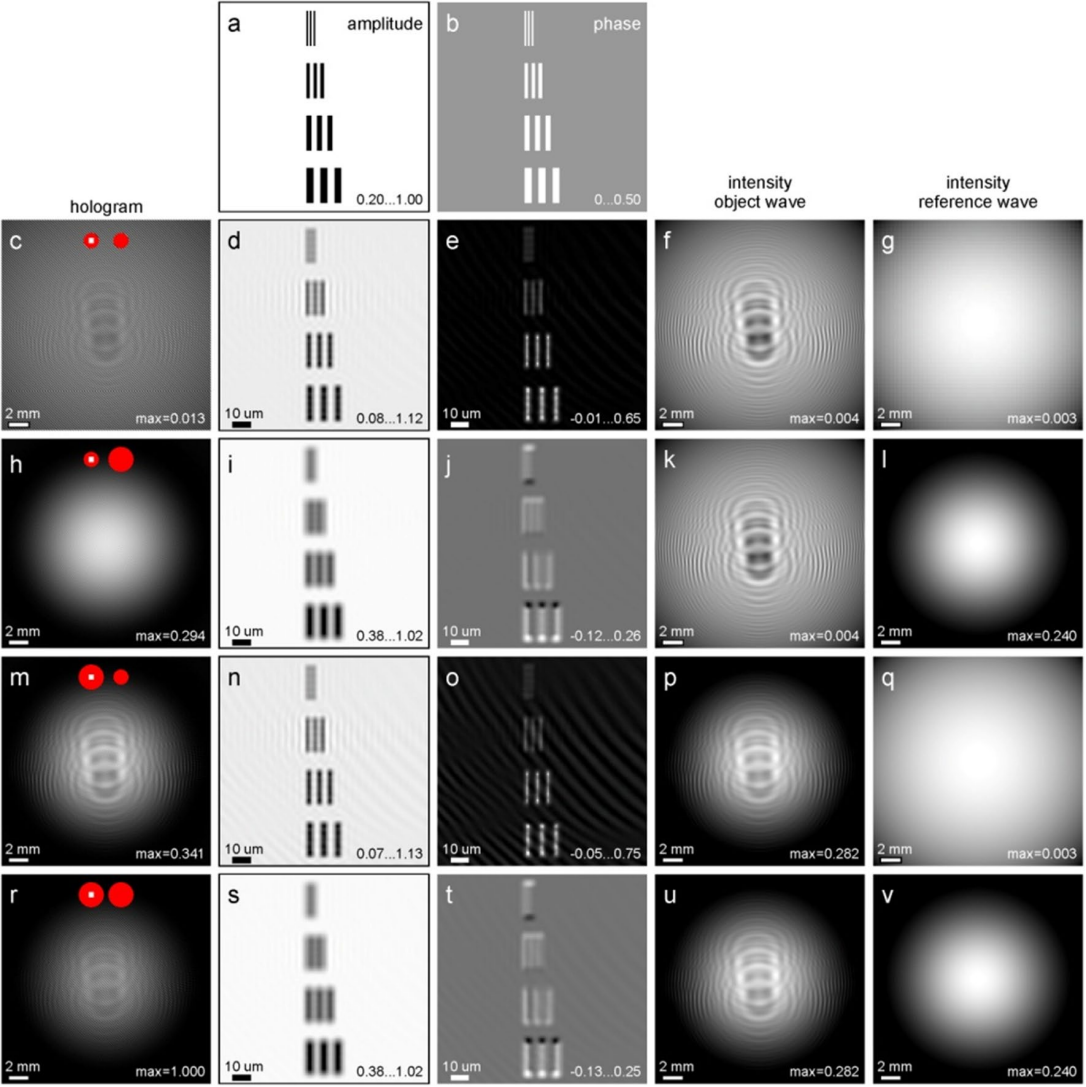
Simulation study of double-slit holography. (**a**) and (**b**) Amplitude and phase distributions of the transmission function in the sample plane. 1st column: simulated holograms. 2nd and 3rd columns: reconstructed amplitude and phase distributions (phase values are in radians), respectively. 4th and 5th columns: Intensity of the object and reference waves separately. The values in the 1st, 4th, and 5th columns indicate the maximal value of the intensity relative to the hologram shown in (r). The diameters of the object and reference apertures are: (**c**)—(**g**) 2 μm and 2 μm, (**h**)—(**l**) 2 μm and 6 μm, (**m**)—(**q**) 6 μm and 2 μm, (**r**)—(**v**) 6 μm and 6 μm, respectively. The red circles in the holograms (**c**), (**h**), (**m**), and (**r**) illustrate the apertures' diameters and the sample position (not drawn to scale).

Simulations were performed to check the effect of the aperture diameters on the resolution and accuracy of the reconstructed amplitude and phase of the sample distribution. The results are shown in Fig. 2. The sample was selected in the form of a series of triplets of bars with 1.0, 2.0, 3.0, and 4.0 μm in both widths and distances between them, with increasing widths from top to bottom rows. The bars were assigned the transmission functions $t\left( {x_{{\text{s}}} } \right) = \exp \left( { - 1.6} \right)\exp \left( {i0.5} \right)$, that is, the absorption coefficient of 1.6, or the amplitude of exp(-1.6) = 0.2 and the phase shift of 0.5 rad. The holograms were simulated as follows. For the object wave, the incident wave in the sample plane was calculated using the analytical expression for diffraction on a round aperture (similar to Eq. (3)), and the propagation to the detector plane was calculated using the same approach as for calculating an in-line hologram created with spherical waves^21^. The wavefront from the reference aperture was calculated using the analytical expression for diffraction on a round aperture (Eq. (5)). The simulation parameters were: λ = 500 nm, *z*_s_ = 2 mm, *z*_d_ = 0.1 m, *D* = 141 μm, and *N* = 1000. The size of the hologram is 50 × 50 mm^2^, the numerical aperture (NA) of the setup amounts to NA = 0.25, and the magnification factor is *M* = *z*_d_/*z*_s_ = 50. The pixel size in the detector plane is 50 μm.

Figure 2 shows the simulated holograms with different diameters of the object and reference apertures, respectively: (c)—(g) 2 μm and 2 μm, (h)—(l) 2 μm and 6 μm, (m)—(q) 6 μm and 2 μm, (r)—(v) 6 μm and 6 μm. The corresponding reconstructed amplitude and phase distributions are also shown. To quantitatively evaluate the mismatch between the modelled $A_{0} \left( {m,n} \right)$ and reconstructed distribution $A\left( {m,n} \right)$, the mean squared error (MSE) was calculated as ${\text{MSE}} = \frac{1}{{N^{2} }}\sum\limits_{m,n}^{N} {\left[ {A\left( {m,n} \right) - A_{0} \left( {m,n} \right)} \right]^{2} }$. The calculated MSEs for the amplitude distributions are: 4.22E-3, 1.00E-2, 4.33E-3, 1.00E-2; and for the phase distributions are: 6.13E-3, 8.64E-3, 8.86E-3, and 9.23E-3; the values are listed for the diameters of the object and reference apertures of: 2 μm and 2 μm, 2 μm and 6 μm, 6 μm and 2 μm, and 6 μm and 6 μm. The following conclusions can be drawn from the results shown in Fig. 2. The best result is achieved when both apertures have the smallest diameter of 2 μm (Fig. 2c–e). In this case, the reconstructed amplitude and phase values agree with the values of the modelled sample. The reconstructions obtained from holograms recorded with both apertures of 6 μm in diameter are of poor resolution and provide inaccurate values of the absorption and phase shifts (Fig. 2r–t). This leads to the conclusion that apertures with smaller diameters provide higher resolution and quality for the sample reconstructions.

Object and reference apertures can also have different diameters. The simulations confirm that the diameter of the reference aperture is more crucial for the resolution and quality of the reconstructed sample than the diameter of the object aperture, as can be seen from comparing the results shown in Fig. 2h–j and Fig. 2m–o.

For all holograms shown in Fig. 2, the resolution evaluated from the parameters of the optical setup amounts to: *R*_NA_ = 1.00 μm (Eq. (12)), pixel size in the sample plane—Δ_s_ = 1.0 μm (Eq. (13)), and the resolution evaluated from the cutout in the Fourier space—*R*_F_ = 2.0 μm (Eq. (14)). However, these resolution criteria do not include the apertures diameters as parameters. The visual inspection of the reconstructed bars shown in Fig. 2 leads to the conclusion that higher resolution is achieved from holograms that were recorded using smaller reference apertures (Fig. 2e and Fig. 2o).

**Figure 3:**
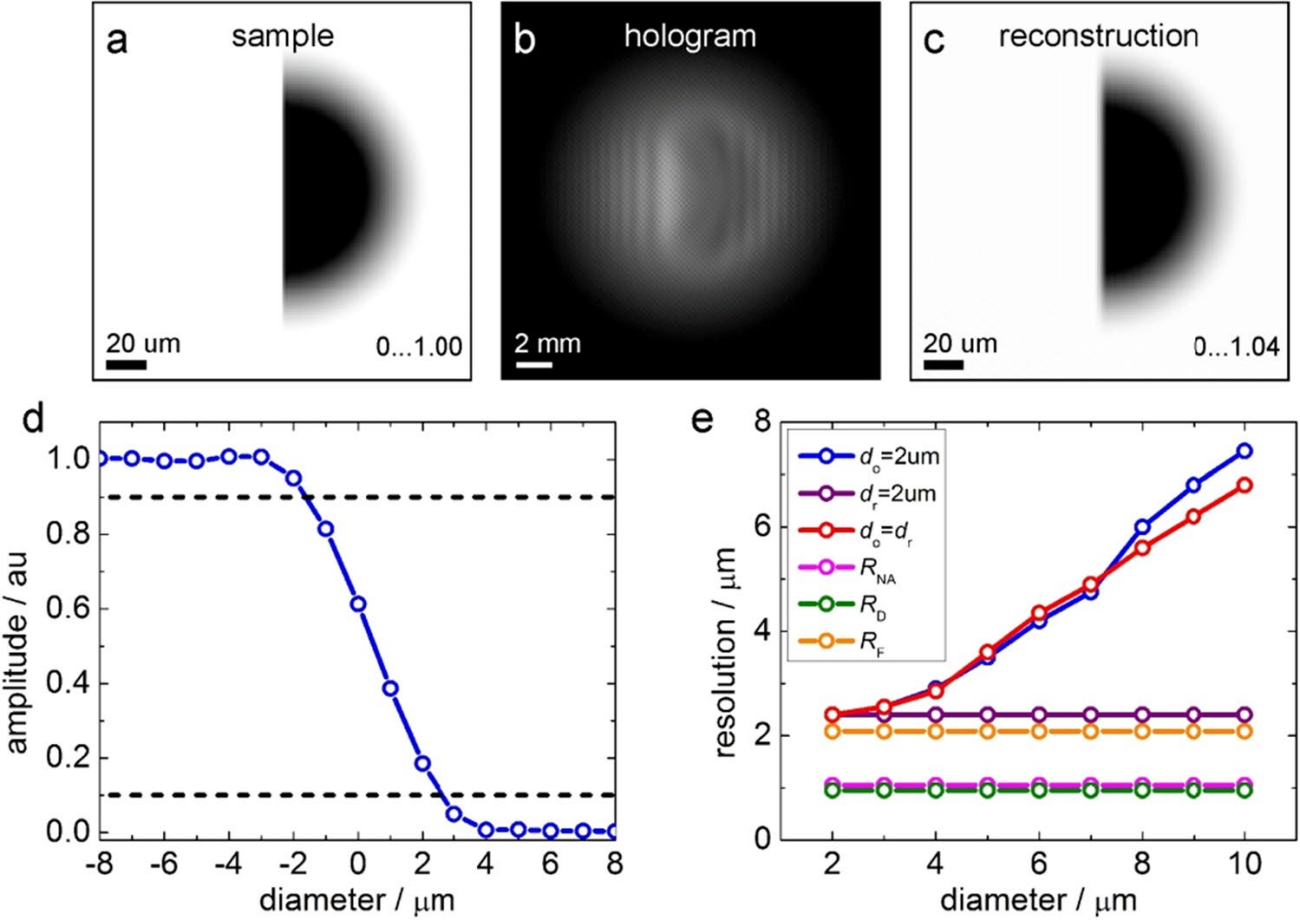
Resolution study (simulations) by using sharp edge object and edge response criterion. (**a**) Amplitude distribution of the transmission function in the sample plane. (**b**) Double-slit hologram simulated using the object and reference apertures of 6 μm diameter. (**c**) Amplitude distribution reconstructed from the hologram shown in (**b**). (**d**) Profile through the middle of the reconstructed amplitude distribution shown in (**c**), which was used to evaluate the resolution according to the edge resolution criterion. The dashed lines indicate 10% and 90% of the maximal amplitude. (**e**) Resolution as a function of the aperture's diameter. The blue curve shows the resolution as a function of the reference aperture's diameter when the diameter of the object aperture remains constant and is 2 μm. The purple curve shows the resolution as a function of the object aperture's diameter when the diameter of the reference aperture remains constant and is 2 μm. The red curve shows the resolution as a function of the aperture diameter when the diameters of the object and reference apertures are the same. The pink curve shows the resolution $R_{{{\text{NA}}}}$, as defined by Eq. (12). The green curve shows the resolution $R_{{\text{D}}}$, as defined by Eq. (13). The orange curve shows the resolution $R_{{\text{F}}}$, as defined by Eq. (14).

The resolution as a function of the diameters of the object and reference apertures was studied in more detail by using a range of the apertures diameters, shown in Fig. 3. As a sample, a sharp edge (amplitude only) was selected (Fig. 3a), so that the resolution can be directly evaluated from the reconstructions of the hologram (Fig. 3b,c) by using the so-called "edge response" criterion^26^ as the distance between 10% and 90% of the maximal amplitude (Fig. 3d). The simulation parameters were the same as in the example above. Simulations were performed for different diameters of the object and reference apertures. The resolution evaluated from the hologram reconstruction, together with the resolution estimated from the parameters of the optical setup (Eqs. (12)–(14) and (19)) are shown in Fig. 3e. The results shown in Fig. 3e, confirm that the resolution is mainly limited by the diameter of the reference aperture. This can be seen from the purple curve in Fig. 3e, which shows the resolution at a constant diameter of the reference aperture (2 μm) and a changing diameter of the object’s aperture: the resolution remains constant and is 2 μm. This resolution is slightly worse than the radius of the reference aperture—1 μm in this case, as given by Eq. (19). This slight mismatch can be explained by the fact that the edge extent used in the edge response criterion can be larger than the extent of the Airy function used in the Rayleigh criterion in Eq. (19).

## Experimental

**Figure 4:**
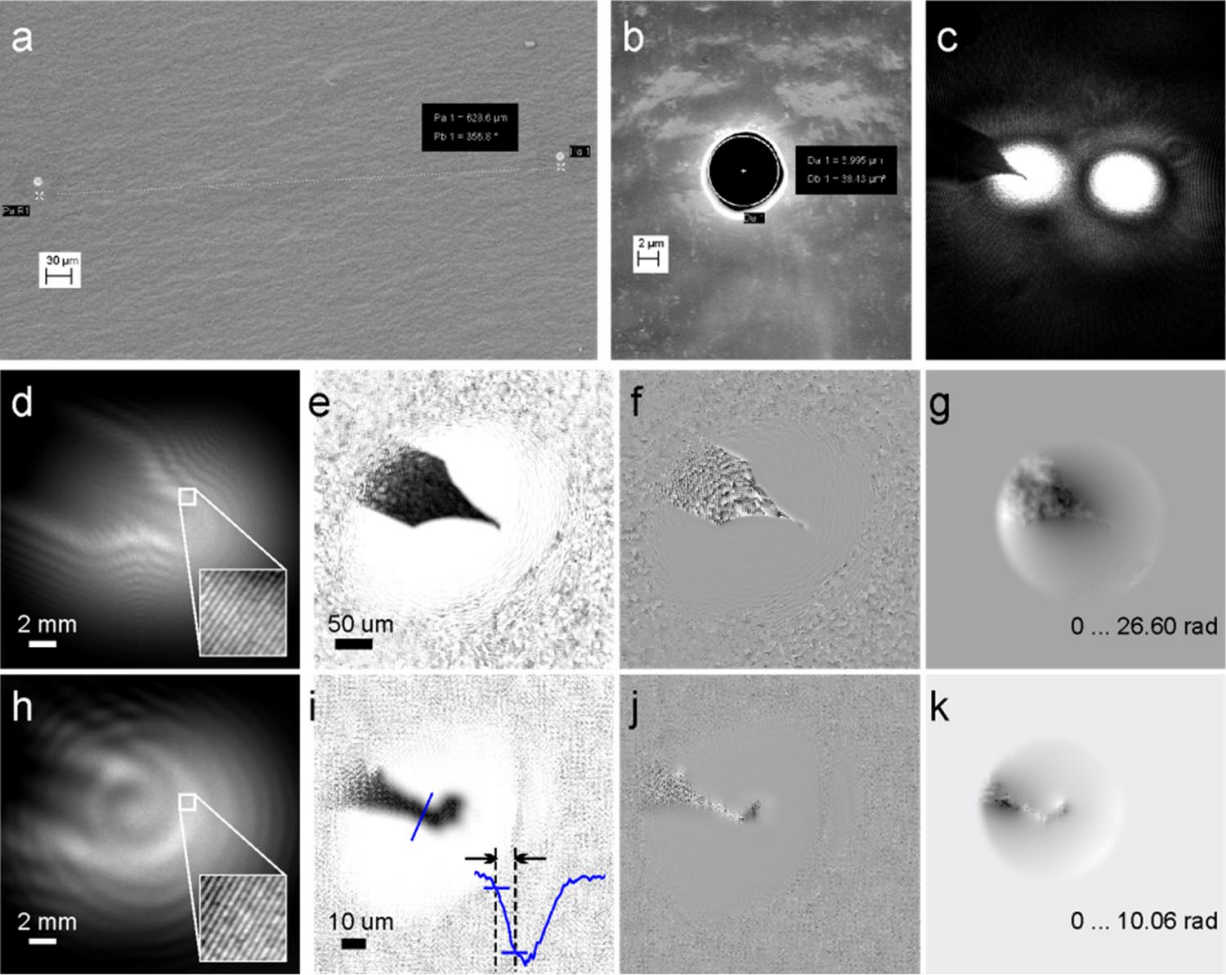
Experimental light optical double-slit holography of a tungsten tip. (**a**) and (**b**) scanning electron microscope images of (**a**) the metal plate with two round apertures (double-slit device) and (**b**) a magnified image of a single aperture. (**c**) A focused light optical image of the sample plane when the object (tungsten tip) is positioned behind the object aperture (here, a different double-slit device with a distance between the apertures of *D* = 190 μm was used). (**d**) and (**h**) holograms recorded at the aperture-to-sample distances of 2.04 and 0.64 mm, respectively; (**e**) and (**i**) the corresponding reconstructed amplitude distributions; (**f**) and (**j**) the reconstructed phase distributions; (**g**) and (**k**) the corresponding unwrapped phase distributions.

Light optical proof-of-concept experiments were performed using 532 nm wavelength laser light. Two apertures of 6.5 μm and 7 μm in diameter and 630 μm apart were fabricated in a thin metal plate by using a focused ion beam; the metal plate was then covered with Aquadag carbon black paint to avoid light reflections. The distance between the double-slit device and the detector was set to 0.095 m. The hologram size was 20 mm × 20 mm. The NA of the setup is thus 0.13. The distance between the sample and the double-slit device was varied to acquire images of the sample at different magnifications. Two holograms were recorded at double-slit device-to-sample distances of 2.04 and 0.64 mm (determined during the reconstruction procedure), respectively. The reconstructed distributions are shown in Fig. 4. The phase unwrapping in Fig. 4g,k was done using the algorithm described elsewhere^27^.

For the holograms recorded at *z*_s_ = 2.04 (0.64) mm, the resolution of the optical setup amounts to (Eq. (12)) *R*_NA_ = 2.53 (2.53) μm, the pixel size in the sample plane is (Eq. (13)) Δ_s_ = 0.43 (0.13) μm, and the resolution evaluated from the cutout in the Fourier space (Eq. (14)) is *R*_F_ = 1.65 (0.52) μm. The resolution given by the radius of the reference aperture is *R*_A_ = 3.5 (3.5) μm (Eq. (19)). Comparing these estimated resolutions, the lowest and therefore the defining resolution is the radius of the reference hole. In addition to the abovementioned criteria, the resolution was evaluated directly from the reconstructions of the experimental holograms (Fig. 4i) using the edge response criterion^26^ as explained above and illustrated in Fig. 3d and Fig. 4i: *R*_edge_ = 4.62 μm, which is slightly worse than the resolution given by the radius of the reference aperture.

## Discussion and conclusions

The proposed single-shot double-slit holography method allows the recording of a hologram that combines the properties of an in-line and off-axis hologram in one single-shot measurement. The method proposed here can be particularly useful for holography with X-rays, where optical elements such as lenses or beam splitters are not available and conventional off-axis holography schemes cannot be realized. Compared to conventional off-axis or previous two-source holography schemes, the proposed double-slit holography has the advantage of using only one single optical element that needs to be placed into the plane wave to create an off-axis hologram. Both apertures are relatively close to each other, which relaxes the requirement for the coherence of the incident wave. Compared to FTH, the proposed method allows imaging at different magnifications by simply adjusting the aperture-to-sample plane distance. The other advantage, when compared to FTH, is that the intensity of the object and reference waves are almost the same when both apertures have comparable diameters. Similarly to FTH, the requirements for the sample are less strict than in in-line holography. Unlike in in-line holography, the imaged object can occupy the entire illuminated area because the reference wave is provided by the reference aperture.

As with any off-axis scheme, the proposed method allows for easy reconstruction of both the amplitude and phase distributions of the sample. The resolution of the reconstructed object is given by the largest value of: the resolution given by the NA of the optical setup, the pixel size in the sample plane, and the radius of the reference aperture. The resolution limitation due to spectral filtering during the reconstruction by cutting out the sideband in the Fourier space (and thus applying a low-pass filter) can be eliminated by using an iterative reconstruction instead of simple Fourier transform sideband selection, which allows to keep the full spectrum of the object wave but only suppresses the zero-order band^28^.

## Data Availability

The datasets used and analyzed during the current study are available from the corresponding author on reasonable request.
